# Exploiting moderate hypoxia to benefit patients with brain disease: Molecular mechanisms and translational research in progress

**DOI:** 10.1002/nep3.15

**Published:** 2023-02-21

**Authors:** Hannelore Ehrenreich, Max Gassmann, Luise Poustka, Martin Burtscher, Peter Hammermann, Anna-Leena Sirén, Klaus-Armin Nave, Kamilla Miskowiak

**Affiliations:** 1 Clinical Neuroscience, Max Planck Institute for Multidisciplinary Sciences, Göttingen, Germany; 2 Institute of Veterinary Physiology and Zürich Center for Integrative Human Physiology, University of Zürich, Zürich, Switzerland; 3 Department of Child and Adolescent Psychiatry and Psychotherapy, University Medical Center Göttingen, Göttingen, Germany; 4 Faculty of Sports Science, University of Innsbruck, Innsbruck, Austria; 5 HBL Investmentpartners GmbH, München-Frankfurt, Germany; 6 Departments of Neurophysiology and Neurosurgery, University of Würzburg, Würzburg, Germany; 7 Department of Neurogenetics, Max Planck Institute for Multidisciplinary Sciences, Göttingen, Germany; 8 Psychiatric Centre, Copenhagen University Hospital, Rigshospitalet, Copenhagen, Denmark; 9 Department of Psychology, University of Copenhagen, Copenhagen, Denmark

**Keywords:** brain EPO circle, erythropoietin, functional hypoxia, HIF, human pilot study, hyperoxia, inspiratory oxygen manipulations, motor-cognitive performance, PBMC, translation

## Abstract

Hypoxia is increasingly recognized as an important physiological driving force. A specific transcriptional program, induced by a decrease in oxygen (O_2_) availability, for example, inspiratory hypoxia at high altitude, allows cells to adapt to lower O_2_ and limited energy metabolism. This transcriptional program is partly controlled by and partly independent of hypoxia-inducible factors. Remarkably, this same transcriptional program is stimulated in the brain by extensive motor-cognitive exercise, leading to a relative decrease in O_2_ supply, compared to the acutely augmented O_2_ requirement. We have coined the term “functional hypoxia” for this important demand-responsive, relative reduction in O_2_ availability. Functional hypoxia seems to be critical for enduring adaptation to higher physiological challenge that includes substantial “brain hardware upgrade,” underlying advanced performance. Hypoxia-induced erythropoietin expression in the brain likely plays a decisive role in these processes, which can be imitated by recombinant human erythropoietin treatment. This article review presents hints of how inspiratory O_2_ manipulations can potentially contribute to enhanced brain function. It thereby provides the ground for exploiting moderate inspiratory plus functional hypoxia to treat individuals with brain disease. Finally, it sketches a planned multistep pilot study in healthy volunteers and first patients, about to start, aiming at improved performance upon motor-cognitive training under inspiratory hypoxia.

## 1 Inspiratory or Functional Hypoxia and the Brain Erythropoietin Circle

Normal neuronal activity depends on adequate tissue oxygenation. Oxygen (O_2_) homeostasis is determined via a balanced O_2_ supply and its consumption by mitochondria. Mitochondrial oxidative phosphorylation utilizes molecular O_2_ as the final electron acceptor to generate adenosine triphosphate (ATP). Hypoxia arises when the cellular O_2_ demand, required to generate sufficient levels of ATP to enable all physiological requirements, exceeds the available supply. Despite its inherent challenge to homeostasis, encompassing numerous mechanisms, hypoxia is frequently encountered and associated with physiological conditions including fetal development or adaptation towards moderate to high altitude. Known cellular environments requiring or experiencing hypoxia include stem cell niches, seminal tubuli, the renal papilla, inflammatory tissue, or the inner mass of solid tumors. Previously interpreted as principally pathological, for instance, upon cardiac arrest, hypoxia is thus increasingly recognized also as an important physiological driving force.^1–15^

In 2019, P. J. Ratcliffe, W. G. Kaelin, and G. L. Semenza jointly received the Nobel Prize in Physiology and Medicine for their pivotal discoveries of how mammalian cells sense and adapt to altering O_2_ availability. A specific transcriptional program, induced by hypoxia, allows cells to acclimate to lower O_2_ levels and/or to limited metabolic support.^1–11,16,17^ The transcription is partly controlled by hypoxia-inducible factors (HIFs) binding to hypoxia-responsive elements to modulate gene expression of potent growth factors such as erythropoietin (EPO)^10,18–28^ and is partly HIFindependent.^10,28^ Amazingly, this same transcriptional program is induced by extensive motor-cognitive exercise during a complex running wheel task in mice and seems to be fundamental for a lasting adaptation of the brain in general and the hippocampus in particular to increased physiological challenge. We have coined the term “functional hypoxia” for this important prerequisite of physicocognitive, demand-responsive “brain hardware upgrade.”^29^

**Figure 1:**
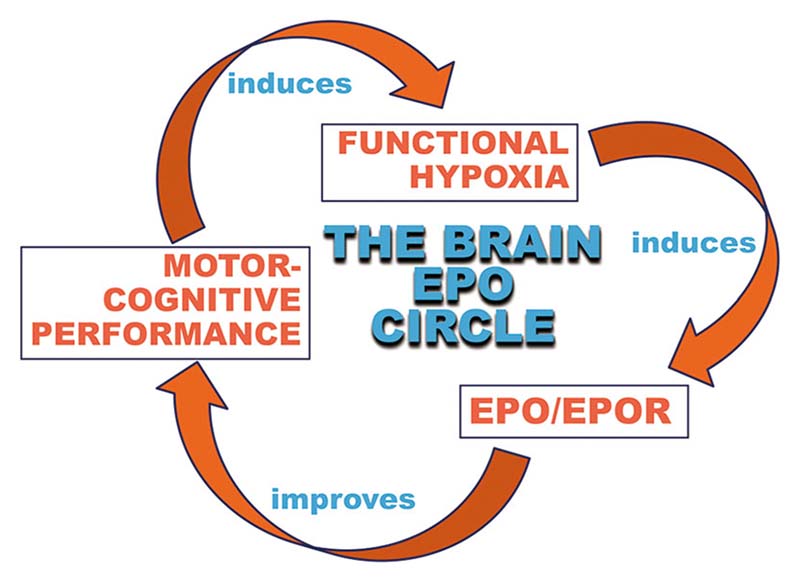
The brain EPO circle. Motor-cognitive activity induces functional hypoxia in the brain. A hypoxia-induced transcriptional program in brain cells includes upregulation of the expression of erythropoietin (EPO) and its receptor (EPOR), which in turn lead to improvement in motor-cognitive performance. This circle can essentially be entered anywhere, starting with inspiratory (instead of functional) hypoxia or with recombinant human (rh)EPO treatment (i.e., exogenous EPO)—both ultimately leading to improved motor-cognitive performance as well.

Extensive physical activity and cognitive challenges are known to lead to widespread brain activation, brain volume increases, and improved global brain function ranging from cognition to mood.^30–32^ We hypothesize that activity-induced, “functional hypoxia” of neurons and brain-expressed EPO play essential roles in all these circumstances in the sense of “brain doping.” Similar effects of a “brain hardware upgrade” are seen upon inspiratory hypoxia.^18,29,33–35^ In other words, strong motor-cognitive activity leads to neuronal activation and functional hypoxia, inducing HIF stabilization, followed by EPO transcription (among other transcripts) in pyramidal neurons, which in turn grow more dendritic spines and simultaneously stimulate their neighboring cells, ready to become neurons, to differentiate within the hippocampus. This brain hardware upgrade includes an EPO induced ~20% increase in pyramidal neurons and oligodendrocytes in cornu ammonis hippocampi, all occurring in the absence of elevated DNA synthesis.^18,36^ In parallel to mediating this novel form of swift adult neurogenesis, EPO reduces microglia numbers and dampens their activity and metabolism as prerequisites for undisturbed neuronal differentiation and maturation.^34,37^ This “brain EPO circle” (Figure 1) contributes to improved brain function including boosted cognition on demand. Application of recombinant human (rh)EPO imitates the effects of brain-expressed EPO, perfectly explaining the strong neuroprotective and procognitive action of this treatment.^29^ Mechanistically, hypoxia-induced EPO (as much as rhEPO) may also have an impact, for example, on regulating oxidative metabolism and mitochondrial function.^38^

Still unfamiliar with the long-recognized brain-expressed EPO system,^39,40^ some critics keep wondering why the “blood hormone” EPO should play such a remarkable protective and procognitive role in the brain. In this context, we propose that evolution has extended an existing and very precise O_2_ sensing system that originally enhanced EPO synthesis for improved motor-cognitive performance, neuroprotection, and neuroregeneration, to increase erythropoiesis in conditions of reduced oxygenation such as blood loss or exposure to altitude. In other words, the original properties of EPO found in species without hematopoiesis have obviously been conserved. Of note, we recently provided evidence that the O_2_ sensing system (prolyl hydroxylase domain protein, PHD2/HIF-2/EPO axis) is very precise and accurate, allowing detection of subtle O_2_ alterations occurring already at low to moderate altitude, that is, between 200 and 2000 m above sea level.^41^ In fact, we observed that every 300 m of altitude increment led to a modest but distinct increase in hemoglobin levels in healthy young men. The question arises as to why humans are equipped with such a precise sensing mechanism to control hematopoiesis. We hypothesize that from an evolutionary point of view, the O_2_ sensing mechanism did not originally evolve to increase red blood cell production by elevating EPO synthesis. For example, mosquitos that do not produce red blood cells are furnished with HIF pathway components including PHD-1.^42^ In addition, the fact that EPO-like genes are found in invertebrates suggests that an EPO-like protein evolved about 550 million years ago^43,44^ and indicates that this O_2_-dependent EPO pathway has been kept and continued to be modified by evolution. Along these lines, rhEPO induced protection and enhanced regeneration of neuronal cells isolated from grasshoppers.^44^

## 2 Exploiting O_2_ Manipulations for Improving Brain Function: Effects of Hypoxia Versus Hyperoxia

We are just starting to understand the effects of O_2_ manipulations on brain performance and brain disease, and how they can potentially be exploited for novel, nonpharmacological treatment approaches. There is a huge amount of literature on purposeful O_2_ manipulations in both sports and the health system. Most papers document the positive or beneficial effects of targeted exposures,^45,46^ while only a few report on potential negative aspects.^47^ Humans have been exposed to various kinds of inspiratory hypoxia or hyperoxia, either in a chronic fashion or in a swiftly alternating way, as applicable via face masks. To make it even more complicated, different conditions for applying air pressure changes were introduced and have to be considered. Whereas exposure to high altitude results in hypobaric hypoxia, the so-called hypoxia chambers allow the application of normobaric hypoxia, and various approaches were taken by researchers, among others in the armed forces, to even apply hyperbaric hyperoxia. In this context, multiple variables, such as the duration, the total number of exposures, the kind of training, and the cessation of exposure, descending from high altitude versus simply stepping out of a chamber, have to be taken into account.^48–54^

From all this work, we learn that the effects and consequences of these variable exposures are obviously diverse, including measured target functions (cognition, motor performance, others), evaluated gene expression levels, for example, from blood cells, or assessed physiological parameters. The data obtained from rodent models or even other species is certainly highly informative, but not always completely translatable to humans, apart from the fact that truly systematic investigations are rare. Drawbacks in published human studies include the highly inconsistent experimental conditions, the small numbers of individuals tested, and the sometimes insufficient exposure time to altered O_2_ levels or too low degrees of O_2_ changes, to just name a few.^54–57^

A whole array of different methods of modulating O_2_ availability has been described to influence brain functions in health and disease. Of note, potential health benefits may not be immediate consequences of hypoxia or hyperoxia per se, but may be a result of adaptations evoked by these stimuli.^58–60^ Although several studies demonstrated favorable health effects associated with living at moderate to high altitudes, that is, hypobaric hypoxia, they include other environmental and socioeconomic factors that are difficult to control.^61–64^ Clearly defined prophylactic or therapeutic effects have been attributed to the use of normobaric hypoxia. In contrast to rather few reports on the application of hypoxia in patients suffering from multiple sclerosis, Parkinson's or Huntington's disease,^55,56,65,66^ there are several studies suggesting beneficial impacts of hypoxia on cognitive performance, dementia, and Alzheimer's disease.^48,67–71^ Moreover, hypoxic exposure has been suggested as a potentially effective treatment for human diseases associated with mitochondrial dysfunction.^72^

Neuroprotection by calibrated hypoxia programs may be fostered by increasing cerebral perfusion and oxygenation, the reduction of cardio- and cerebrovascular risk factors, for example, systemic hypertension, dyslipidemia, and glucose intolerance, the upregulation of neuroprotectants, for example, EPO, vascular endothelial growth factor (VEGF), nitric oxide, and/or antioxidants, but also by suppressing neuronal apoptosis.^73–75^

Hyperoxia, the rise in inspiratory O_2_, is never physiological, but represents an artificial, rather pharmacological intervention. It increases brain oxygenation as O_2_ easily diffuses across the blood–brain barrier and may possess therapeutic potential after, for example, brain injuries.^76,77^ In fact, the exclusive use of hyperoxia looks back on a long tradition in the clinical therapy of illnesses characterized by hypoxemia.^78^ For example, normobaric hyperoxia represents a principle measure in the acute management of circulatory shock, especially in trauma patients,^79^ but is also the application of choice in hypoxemic respiratory failure to be treated in the intensive care unit.^80^ It has been suggested as a simple and widely accessible therapeutic strategy, associated with improved clinical outcomes in stroke patients.^81^ Beneficial effects of normobaric hyperoxia for brain protection are considered to primarily result from improvements in brain metabolism and the resistance to cell damage.^82,83^ Moderate normobaric hyperoxia (10×2h at 37% O_2_) in healthy volunteers apparently improves cognitive performance and increases blood levels of antioxidant enzymes and neurotrophic factors.^84^ Advances in normobaric hyperoxia application, particularly derived from animal studies evaluating effects in experimental stroke and brain trauma, have recently been reviewed.^85^

While normobaric hyperoxia is commonly used in the settings mentioned above, the use of hyperbaric O_2_ therapy seems to be more effective in other circumstances, for example, acute severe traumatic brain injury,^86^ or carbon monoxide poisoning.^87^ Noteworthy in this context, in severe burns, beneficial effects of hyperbaric O_2_ therapy may include the attenuation of central sensitization.^88^ Further examples are recent work on hyperbaric O_2_ therapy that reported alleviation of vascular dysfunction and amyloid burden in an Alzheimer’s disease mouse model and also in elderly patients.^89^

Similarly, intermittent hypoxic–hyperoxic training in geriatric subjects^67^ and patients with mild cognitive impairment^48^ showed beneficial effects on cognition. Intermittent hypoxia or hypoxia–hyperoxia conditioning programs commonly apply multiple exposures to hypoxia (3–8 min, 10%–16% O_2_), interspersed by brief (2–5 min) exposures to normoxic (21% O_2_) or hyperoxic (30%–40% O_2_) conditions; with a total exposure duration of 30–40 min per session; applied at 1- or 2-day intervals over 2–8 weeks.^48,67,90–93^ Findings from preclinical and clinical experiments provide valuable hints on the neuroprotective potential of such intermittent programs.^73,94,95^

The combined use of hypoxia and hyperoxia might evoke complementary and therapeutically more favorable effects than either one alone, consistent with sort of a hyperoxic–hypoxic paradox.^58,96^ This is supported by recent intermediate interventions,^48,67^ with benefits likely resulting from overlaps of gene transcription programs, for example, HIF (see also below) or an emerging regulator of cellular resistance to oxidants, nuclear factor erythroid 2-related factor 2 (Nrf2), inducing more robust adaptive processes than hypoxia or hyperoxia alone.^58,94^

Also, upon chronic moderate normobaric hyperoxia (50% O_2_ for 3 weeks), increased stabilization of the α-subunits of HIF-1 and HIF-2, as well as elevated expression of VEGF and EPO has been reported in the mouse brain,^97^ pointing to some overlap in downstream mechanisms of hypoxia and hyperoxia, reflected or mediated, for example, by excess reactive oxygen species.^96,98,99^ Moreover, underlying molecular mechanisms include variable changes in the expression of caspase-3, matrix metalloproteinase-9, aquaporin-4, and Na+/H+ exchanger-1.^85,100,101^

On the other hand, contrasting effects of hypoxia versus hyperoxia were found, for example, in a mouse model of Friedreich's ataxia where breathing of 11% O_2_ attenuated the progression of ataxia, whereas breathing 55% O_2_ hastened it.^102^ Effects of hypoxia versus hyperoxia on gene expression and functional or structural neuroplasticity have not been studied yet systematically and back-to-back. Pilot data from our laboratories indicate that this will be essential to understand and exploit the underlying mechanisms. In preliminary work, using a transgenic reporter of transient hypoxia (CaMKIIα-CreERT2-ODD::R26R-tdTomato mice, expressing the HIF-1α oxygen-dependent degradation-domain, ODD, fused to CreERT2-recombinase^103^ for persistent activation of a fluorescent reporter after hypoxia), we surprisingly found a mild increase in red-labeled (tdTomato+) neurons also after exposure to inspiratory hyperoxia (unpublished observations). It is unclear, however, whether this was due to the stabilization of CreERT2-ODD also at high O_2_ concentration or to a rapidly sensed “relative hypoxia” after cessation of hyperoxia. Therefore, using, for example, sophisticated ultrastructural analyses,^104–106^ we are presently testing the hypothesis that the intracellular mechanisms of hypoxia and hyperoxia are in part similar, affect functional and structural neuroplasticity, and involve the activation of EPO/EPOR signaling in the brain. In fact, synaptic transmission is highly energy demanding relying on oxidative metabolism.^107^ Excitability of hippocampal CA1 pyramidal cell synapses, for instance, is dependent on O_2_ tension.^108^ Plasticity-associated proteins such as presynaptic calcium channels and active zone scaffolds as well as postsynaptic glutamate receptors are likely candidates to be altered upon hypoxia or hyperoxia. Indeed, structural changes in hippocampal CA1 synapses after hypoxia resemble activity-dependent plasticity, including calcium and NMDA receptor-dependent remodeling of postsynaptic spines and the formation of presynaptic filopodia.^109,110^ Putative modifiers of structural plasticity may include O_2_ sensors such as HIF-1/ HIF-2^97^ and PHD2,^111^ as well as nitric oxide^110^ and growth factors like brain-derived neurotrophic factor (BDNF)^112^ and EPO.^18,33,97^

## 3 Moderate Inspiratory Hypoxia Plus Motor-Cognitive Training to Treat Patients with Brain Disease: Where we are and Where we are Going

In contrast to acute mountain sickness, usually observed after ≥6 h of exposure to hypobaric hypoxia,^113^ it has been known for many years from sports medicine and altitude research that short and moderate inspiratory O_2_ manipulations are well tolerated by human subjects and can lead to improved performance. However, truly systematic studies to understand the effect under strictly defined conditions are widely lacking. In particular, controlled hypoxia studies in patients are still rare and highly heterogeneous in terms of type (O_2_ concentration under normo-, hypo- or hyperbaric conditions), application (hypoxia chamber, generators with masks), and respective duration of inspiratory O_2_ manipulations, and usually include only a few subjects and often no proper controls. In addition, there is frequently a lack of monitoring and follow-up with convincing parameters/ biomarkers of success. Some studies show that EPO is elevated in blood under these conditions.^114,115^ This is also in agreement with our own studies in mice, which, moreover, show that a significant increase in EPO expression in the brain can be expected even with short exposure to hypoxia for a few hours.^33,34^

We are presently preparing for systematic studies to investigate the effect of inspiratory hypoxia on human motor-cognitive performance. In parallel, we aim to carefully examine human blood cells for their hypoxia response. In fact, HIF-dependent changes in metabolism profoundly affect the phenotype and function of immune cells.^116^ A new fluorescent antibody cell sorting device with thus far unique possibilities to characterize and isolate human blood cell subpopulations is now up and running in one of our laboratories (HE; BD FACSymphony™ S6 cell sorter with 7 lasers). Blood cell studies include the determination of relative numbers and cell types, but also single-cell or nuclei transcriptome analyses. These in turn will help select transcripts that might be useful for response prediction. To achieve this goal, translational approaches from rodents to humans are planned. Healthy wild-type mice will—analogously to humans (see below)—either be exposed for 3 weeks to daily 3.5 h of hypoxia (90 min down from 16% to 12%; then 2 h at 12%) in a rodent hypoxia chamber or serve as normoxia controls. Immediately after the last exposure, brain (cortex, hippocampus, cerebellum, brainstem) and peripheral blood mononuclear cells (PBMCs) will be prepared for single-cell/nuclei RNA-sequencing as described.^18,33^ This will allow the identification of PBMC transcripts in mice that correlate with the observed response of brain regions/cell types to hypoxia. We expect sufficient overlap of intraspecies PBMC and brain transcripts^117^ to approach interspecies analyses next. Identified PBMC markers that correspond to the brain hypoxia response in mice will be used to infer the human brain response from human hypoxia-induced PBMC gene expression. Trans-species analogously hypoxia-stimulated transcripts in the brain (mouse) and PBMC (mouse and human) will be identified in health, and later exploited for disease. Novel bioinformatic tools of small conditional RNA-sequencing data analyses will—based on these data—allow the generation of various prediction models of a beneficial (e.g., procognitive, regenerative, remyelinating, or anti-inflammatory) response also in humans to moderate hypoxia.^118–122^ This will also include effects on energy metabolism,^116^ which is part of the glial support of neuronal network functions.^123,124^ Astrocytes are a local source of lactate for glutamatergic synapses,^125^ and myelinating oligodendrocytes have a similar function when metabolically supporting spiking axons with lactate/pyruvate.^126–128^ Thus, the bulk of neuronal ATP production in the brain is mitochondrial oxidative phosphorylation, whereas the lactate-producing glial cells operate at least in part by aerobic glycolysis.^126,129^ The major function of HIF is the adjustment of the organism to hypoxia, which includes the upregulation of genes for glucose import and glycolysis. In cancer cells which—similar to glial cells —switch to aerobic glycolysis (*Warburg effect*), the hypoxia-induced enzyme pyruvate kinase M2 even serves as a feedforward transcriptional coactivator for HIF-1 expression/stabilization.^130^ Thus, with respect to the cellular energy balance under functional hypoxia, the critical question arises whether a possible dampening effect on mitochondrial respiration is outweighed by an upregulation of glycolytic ATP and lactate production. Interestingly, combined functional magnetic resonance imaging and ^15^O positron emission tomography (PET) imaging studies in humans showed, for the visual system, that the neurovascular responses to neuronal activity are not closely coupled to the actual decreases of tissue oxygenation and O_2_ requirements.^131,132^ Thus, it is plausible that functional hypoxia at the cellular level can increase glucose consumption and overall energy production in a HIF-dependent fashion well before a lack of O_2_ puts a break on overall ATP production. Here, the influence of hypoxia-induced EPO on mitochondrial function may play a crucial role.^38^

To test mild inspiratory hypoxia together with functional hypoxia, induced by physicocognitive exercise, as a synergistic approach in humans, hypoxia training chambers (HÖHENBALANCE GmbH, Going, Austria) with floor areas of approximately 16–20 m^2^ have been installed in our institutes, that is, thus far the MPI-NAT outpatient research clinic (HE) and the Department of Psychology, University of Copenhagen (KM). The chambers are equipped with computer desks, set up for cognitive training (Happyneuron, Humansmatter, Lyon, France), as well as bicycle ergometer and treadmill (h/p/cosmos sports & medical GmbH, Nussdorf-Traunstein, Germany). The physical training devices will have large screens in the front that can be used to apply entertaining movies, keeping test subjects stimulated for workouts, or even cognitively challenging online tests, thus allowing simultaneous motor-cognitive challenges. Comprehensive online data monitoring will be professionally performed and supervised to allow wide-ranging structured analyses (Datico Sport & Health GmbH. Burghausen, Germany).

## 4 Pilot Study on Motor-Cognitive Training Under Inspiratory Hypoxia about to Start: Multistage Procedure Planned

Due to the still exploratory nature of the planned project, the following pilot study is launched, which builds on two decades of own basic and clinical EPO (H. E., M. G., A. L.-S., K.-A. N., and K. M.) and high-altitude research (M. G. and M. B.) and with which first own experience with the hypoxia chamber can be acquired in a multistep procedure:

(1) In the first step, the researchers themselves will— as healthy volunteers—test the altitude training chamber, and initially experience different O_2_ concentrations for different periods of time up to several hours daily. At the same time, the O_2_ saturation in the blood is closely monitored using pulse oximeters. After convenient adaptation to the reduced O_2_ concentration in the atmosphere, the first physical and cognitive training sessions under hypoxia are offered. For this purpose, the training devices (ergometer, treadmill) can be combined with neuropsychological tasks that are communicated via screens. Most importantly, however, probands can choose to sit at a computer desk for intensive cognitive training. This first step aims at free exploration and has no fixed schedule. Subjects are just encouraged for an optimal outcome to practice using all three alternative options as much as they can. The observations will then feed into step 2.
(2) In the next step, 8–10 healthy volunteers will, after adequate habituation, be exposed to 3.5 h of hypoxia daily over the course of 3 weeks. The researchers assume that healthy adult subjects will show improvements in cognition and performance as active participants in the training chamber. The subjects enter the normobaric chamber, which is stably set to 16% O_2_. Then, the O_2_ is slowly reduced within 90 min and replaced by nitrogen until 12% O_2_ is reached. The duration of this habituation phase is anticipated to last from days 1 to 3 of the 3-week experiment, with some expected inter-individual differences in convenience and speed of adaptation. This has to be tested individually and optimal conditions will have to be compiled. Ultimately, probands should enter the chamber at 16%, experience a decrease to 12% within 90 min, and keep training under 12% O_2_, which continues for 2 h daily over 3 weeks. Its intensity, that is, the frequency of ergometer or treadmill use, can be decided by each participant during this exploration phase of the pilot study, that is, is left up to the individual and should be done according to motivation and well-being, but will be recorded very precisely. Later, a standardized plan will be derived from this information.
(3) In case of success, that is, hints of the expected intraindividual improvement of cognition and motor performance of volunteers, and good tolerability, first patients suffering from multiple sclerosis, chronic schizophrenia, or autism will be allowed to undergo a similar procedure in the sense of compassionate use approaches after intensive informed consent. Patients are already waiting to participate (approximately 8–10 subjects per diagnosis will ultimately be recruited). Exclusion criteria include, for example, heart or lung disease and epilepsy.
(4) In case of a positive outcome of this pilot study, that is, a measurable intraindividual improvement in function, selected parameters will be specifically and systematically changed in a series of subsequent steps. These include, for example, the exposure time: Can it be shorter? The training mode: How much training is most helpful? Follow-up examinations of cognition and motor function (as well as blood cells) over a few weeks to months after completion of the 3-week hypoxia training are intended, too.

Throughout the entire period of the pilot study, the researchers will be consulted and guided by an advisory board of experts (altitude researchers, sports physicians, cardiologists) providing advice and exchange. Interdisciplinary and international cooperations (e.g., with colleagues in Harvard, New York, Innsbruck, Zürich, Cambridge, Oxford, etc.) are already being initiated. The first publishable results are expected in about 1–2 years after the start. Based on this preliminary work, the ERC Consolidator Project ALTIBRAIN (awarded to K. M., with HE as the collaborator) will start soon. In parallel studies in healthy individuals, patients with affective disorders and mice, we will investigate whether intermittent moderate hypoxia (12%) combined with motor-cognitive training over 3 weeks is sufficient to enduringly increase cognitive performance and hippocampal volume and function, as well as the maturation of neural progenitor cells and dendritic spines. This will be measured (1) in humans with PET, using synaptic vesicle glycoprotein 2A UCB-J binding to reflect dendritic spine density, and (2) in mice (which also allows determining the induced expression of brain EPO) with immunohistochemistry, for example, Golgi staining and Light sheet microscopy. The findings can lead to a breakthrough in the understanding of mechanisms underlying enduring neuroplasticity and to novel treatment strategies targeting cognitive decline.

Moreover, our planned “high-altitude” training of autistic children and their parents in our hypoxia chamber will commence in due course (L. P. with H. E.). This is a worldwide unique pilot study in a frequent condition, challenging diagnostic competences as well as reliability of follow-up measures,^133,134^ and thus far lacking biology-based treatments.^135^ We hypothesize that intensive motor-cognitive training within a defined social setting (family condition) that consists of 3 h daily for 3 weeks in a hypoxia training chamber (12% O_2_; conditions comparable as described above for adults) will lastingly improve autistic symptoms and motor performance as well as improve cognitive dysfunction in children diagnosed with autism and/or intellectual disability. Among the principal mediators of the beneficial hypoxia effects, we see, in particular, the hypoxia-induced brain EPO system. We expect that this early intervention in a period of still the highest brain plasticity will enable enduring amelioration or substantial corrections of developmental trajectories at risk. This requires developmental alterations to be partly reversible, which was actually confirmed in models of syndromic ASD, that is, *Syngap1* haploinsufficiency and Rett syndrome,^136,137^ as well as in early intervention studies.^138^ Parents (as concurrently challenged adults in the chamber) will show profits for their own cognition and general performance. Moreover, we expect advantageous effects also for the parent–child interaction and synchrony as another central feature of favorable outcome and prognosis. We anticipate that the beneficial outcome will overall exceed that of identical training conditions under normoxia.

Based on these studies, it will eventually be possible to initiate the planning of larger trials, which will then have realistic chances of receiving appropriate financial support from public funds. If successful, requests for follow-up studies will emerge quickly due to the considerable worldwide need for disease-modifying therapies for brain diseases. Since most likely, the pharmaceutical industry may not be too much interested in nonpharmacological approaches, new financing strategies and ideas will have to be pursued. Only dissemination and potential economic exploitation will allow broader accessibility of these nonpharmacological treatments to the healthcare system and general public. At the same time, effective replication studies and multicentric endeavors for scientific consolidation of treatment success demand standardized and highly comparable methodological approaches. It may be worthwhile even considering *franchising* as a means of expanding and protecting successful protocols.

The various expected positive impacts of inspiratory and functional hypoxia on brain function but also other physiological parameters might deliver a broad variety of potential health benefits. Setting the scene for applications via structured and scientifically assessed trials using modern methodologies of digital data processing and sophisticated laboratory testing can improve the convergence of scientific results also into economically attractive treatment protocols as prerequisite of translatability into the real world. Differentiated knowledge regarding hypoxia effects on human performance may lead to more effective treatment procedures. Even after acute events like, for example, stroke, with the necessity of rapid invasive pharmacology, subsequent hypoxia training may prove efficient for recovery, as well as cost-saving with lower side effects. Respective therapeutic offers might later be aligned with health, vacation, or rehabilitation centers, for instance, in the mountains or in favorable seawater environments. In this sense, also prevention could gain increasing significance.

## Data Availability

Not applicable.

## Highlights

- This review focuses on the brain and sketches hypoxia as a physiological driving force, inducing specific transcriptional programs.
- Moderate inspiratory hypoxia may improve brain function and performance.
- Our concept of “functional hypoxia” is introduced as a demand-responsive mediator of “brain hardware upgrade” on extensive motor-cognitive exercise.
- Hypoxia-induced erythropoietin (EPO) expression in the brain plays a decisive role in these processes, constituting what we coined the “brain EPO circle.”
- The brain EPO circle underlies the adaptation to challenge, that is, enhanced brain function including cognition and accomplishment on demand: “Brain doping.”
- Recombinant human EPO imitates the effects of brain-expressed EPO, explaining the strong neuroprotective and procognitive impact of this treatment.
- A pilot study in healthy volunteers and first patients is outlined, exploiting moderate inspiratory plus functional hypoxia in humans for improving cognition.
